# Natural Killer Cells Mediate Protection against *Yersinia pseudotuberculosis* in the Mesenteric Lymph Nodes

**DOI:** 10.1371/journal.pone.0136290

**Published:** 2015-08-21

**Authors:** Maik Rosenheinrich, Wiebke Heine, Carina M. Schmühl, Fabio Pisano, Petra Dersch

**Affiliations:** Department of Molecular Infection Biology, Helmholtz Centre for Infection Research, Braunschweig, Germany; Emory University School of Medicine, UNITED STATES

## Abstract

Natural killer cells play a crucial role in the initial defense against bacterial pathogens. The crosstalk between host cells infected with intracellular pathogens and NK cells has been studied intensively, but not much attention has been given to characterize the role of NK cells in the response to extracellular bacterial pathogens such as *yersiniae*. In this study we used antibody-mediated NK cell depletion to address the importance of this immune cell type in controlling a *Y*. *pseudotuberculosis* infection. Analysis of the bacterial counts was used to follow the infection and flow cytometry was performed to characterize the composition and dynamic of immune cells. Depletion of NK cells led to higher bacterial loads within the mesenteric lymph nodes. We further show that in particular CD11b^+^ CD27^+^ NK cells which express higher levels of the activation marker CD69 increase within the mesenteric lymph nodes during a *Y*. *pseudotuberculosis* infection. Moreover, in response to the activation NK cells secrete higher levels of IFNy, which in turn triggers the production of the proinflammatory cytokine TNFα. These results suggest, that NK cells aid in the clearance of *Y*. *pseudotuberculosis* infections mainly by triggering the expression of proinflammatory cytokines manipulating the host immune response.

## Introduction

The genus *Yersinia* includes three species, which are well known to cause infections in humans: *Yersinia pestis*, *Y*. *pseudotuberculosis* and *Y*. *enterocolitica*. *Y*. *pseudotuberculosis* and *Y*. *enterocolitica* are enteric pathogens associated with food borne infections resulting in different intestinal diseases such as diarrhea, enteritis and mesenteric lymphadenitis addressed as Yersiniosis [1, 2]. In immunocompromised individuals the inability to control the infection and to limit inflammation can lead to severe sequelae such as erythema nodosum and reactive arthritis [1]. *Y*. *pestis*, a close relative of *Y*. *pseudotuberculosis*, is transmitted by rodents and infected fleas, and causes severe illness (bubonic and pneumonic plague) and death in humans [3].

All three *Yersinia* species are characterized by their tropism for lymphatic tissues [4]. After colonization of the gastrointestinal tract by both enteric *Yersinia* species, the bacteria invade into underlying lymphatic tissue, the Peyer´s patches (PPs) [5]. Subsequently, the bacteria disseminate to the draining mesenteric lymph nodes (mLNs) and reside preferentially in the B- and T-cell zones [4]. Colonization of the lymphatic tissues is accompanied with the initiation of inflammatory processes, including the production of proinflammatory cytokines and the recruitment of phagocytes to the site of infection [6]. In order to counteract this immune response, *yersiniae* produce a set of effector proteins (*Yersinia* outer proteins—Yops) that are translocated into host cells via the type III secretion system (T3SS). All genes encoding the needle-like structure—the injectisome—and the injected Yops are located on a virulence plasmid present in all human pathogenic *Yersinia* strains [7, 8]. The T3SS delivers effector proteins YopE, YopH, YopP/J, YopM, YopO, and YopT into the host cell cytosol where they influence multiple signaling events that prevent phagocytosis, manipulate host immune responses and induce cell death [9].

Innate immunity is an integral part of the response to invading bacterial pathogens. It involves different cell types such as macrophages, dendritic cells (DCs) and natural killer (NK) cells as well as non-cellular components (cytokines, chemokines). NK cells are mainly known to be involved in regulation of tumor growth due to their ability to lyse certain tumor cells [10, 11]. Additionally, NK cells can inhibit the spread of viral infections via their cytolytic action against infected cells and the production of proinflammatory cytokines and immune modulators such as interferon gamma (IFNγ) and the tumor necrosis factor alpha (TNFα) [12, 13]. Strong evidence also exists for the contribution of NK cells to the host immune response to bacterial infections. However, their role and impact on host protection is often unknown or controversial since depending on the experimental model both beneficial and deleterious effects have been attributed to NK cells [14].

Activation of NK cells has been reported for a number of bacterial pathogens and occurs mainly by (i) priming through IL-2 and IL-15 secreted by bystander cells (e.g. DCs, T cells), (ii) secretion of IL-12 and IL-18 by infected macrophages and DCs that sensed bacterial products, and (iii) direct contact between NK cells and the infected phagocyte [12, 13].

Since activated NK cells act predominantly via the production and release of IFNγ, which was shown to protect mice against *Y*. *enterocolitica*, *Y*. *pseudotuberculosis* and *Y*. *pestis* [15–17], we hypothesized that NK cells play a role in clearing *Yersinia* infections. Consistent with this assumption is a report demonstrating that a decrease of NK cells enhanced colonization of *Y*. *enterocolitica* in the spleen [18], but the contribution of NK cells to the host immune response against a *Y*. *pseudotuberculosis* infection remains unknown. In the present study, we employed antibody-mediated NK cell depletion to investigate the role of NK cells in the clearance of a natural oral infection of *Y*. *pseudotuberculosis*. The analysis of the dynamics and activity of different NK cell subsets during the infection revealed that NK cells mediate protection against *Y*. *pseudotuberculosi*s in the mLNs via modulation of cytokine production.

## Materials and Methods

### Ethics Statement

Seven-week-old female C57BL/6J were purchased from Janvier (Le Genest Saint Isle, St Berthevin Cedex, France) and housed under specific pathogen-free conditions according to FELASA recommendations in the animal facility of the Helmholtz Centre for Infection Research, Braunschweig. Animal experiments were carried out in strict accordance to German Recommendations of the Society of Laboratory Animal Science (GV-SOLAS) and the European Health Recommendations of the Federation of Laboratory Animal Science Associations (FELASA). The animal protocols were approved by the Niedersächsisches Landesamt für Verbraucherschutz und Lebensmittelsicherheit: animal licensing committee permission no. 33.9.42502-04-13/1166. Animals were handled with extensive care and all efforts were made to minimize suffering.

### Mouse infection experiments

*Y*. *pseudotuberculosis* strain YPIII [19] was grown at 25°C overnight in LB broth (BD Bioscience), washed and resuspended in PBS. Animals were deprived of food for 12–16 hours prior to infection and infected intragastrically with a ball-tipped needle with 2 x 10^7^ colony forming units (CFUs) of bacteria. Bacterial burden of infected organs was measured as follows: three days after infection, mice were sacrificed via CO_2_ asphyxiation, organs were collected, weighed and homogenized. Serial dilutions of the organ homogenates were plated on LB plates containing 1 μg/ml Triclosan (Calbiochem) to determine the CFUs.

### Antibody-based depletion of NK cells

NK depletion was achieved by intra-peritoneal injection of mice with 100 μg anti-NK1.1 antibody (clone PK136) in 170–200 μl of H_2_O (depending on the weight of the mice) 24 hours prior to infection [20].

### Flow cytometry

Three days after infection, mice were euthanized and mLNs were isolated. Single cell suspensions were generated in PBS supplemented with 0.2% BSA using cell strainers (100 μm, BD Biosience). Cells were counted using a Z2 Coulter Counter (Beckman Coulter). Subsequently, 3 x 10^6^ cells were surface-stained with the following antibodies: Live/Dead Fixable Blue Dead Cell Stain (Invitrogen), CD3:v450 (BD Biosciences, cl.17A2), Ly6G:PE-Cy7 (eBioscience,cl.1A8), CD16/CD32 (BD Biosciences, cl.93), CD4:PerCp-Cy5.5 (eBioscience, cl.RM4-5), CD8a:PE (eBioscience, cl.53-6.7), CD11b:ef450 (eBioscience, cl.M1/70), CD11b:Brilliant Violet 605 (Biolegend, cl.M1/70), CD11c:APC-ef780 (eBioscience, cl.N418), F4/80:PE (eBioscience, cl.BM8), CD19:FITC (eBioscience, cl.N418), CD27:APC-Cy7 (eBioscience, cl.4G.7F9), CD69:PE (eBioscience, cl.H1.2F3), CD86:Pacific Blue (Biolegend, cl.GL-1), CD107a:FITC (Biolegend, cl.1D4B), NK1.1:PE-Cy7 (eBioscience, cl.PK136), Ly6C:APC (BioLegend, cl.HK1.4), CD3e:Biotin (BD Biosciences, cl.145-2C11), CD19:Biotin (eBioscience, cl.eBio1D3), CD49b:Biotin (Biolegend, cl.DX5), Streptavidin:FITC (BD Bioscience), MHCII:PerCP-eFluor 710 (eBioscience, cl.AF6-120.1). Cell subsets were defined as following: B cells (CD19^+^ CD3^-^), T cells (CD19^-^ CD3^+^), cytotoxic T-cells (CD3^+^ CD8^+^), T_H_ cells (CD3^+^ CD4^+^), NK cells (CD19^-^ CD3^-^ NKp46^+^), NKT cells (CD3^+^ NK1.1^+^), neutrophils (CD19^-^ CD3^-^ CD49b^-^ Ly6G^+^ CD11b^+^), macrophages (CD19^-^ CD3^-^ CD49b^-^ Ly6G^-^ CD11b^+^ F4/80^+^), monocytes (CD19^-^ CD3^-^ CD49b^-^ F4/80^-^ CD11c^-^ Ly6G^-^ Ly6C^-^ CD11b^+^), inflammatory monocytes (CD19^-^ CD3^-^ CD49b^-^ F4/80^-^ CD11c^-^ Ly6G^-^ Ly6C^+^ CD11b^+^), DCs (CD19^-^ CD3^-^ CD49b^-^ F4/80^-^ CD11c^+^) and pDCs (CD19^-^ CD3^-^ CD49b^-^ F4/80^-^ CD11c^-^ Ly6G^-^ Ly6C^+^ CD11b^-^).

For intracellular staining 3 x 10^6^ cells were stimulated for three hours using Cell Stimulation Cocktail plus protein transport inhibitors, including phorbol 12-myristate 13-acetate (PMA), ionomycin, brefeldin A and monensin (eBioscience). Cells were stained with surface antibodies and permeabilized using BD Cytofix/Cytoperm (BD Biosciences) according to the manufacturers recommendations. Subsequently, intracellular staining was performed with the following antibodies: IFNγ:PerCP-Cy5.5 (eBioscience, cl.XMG1.2), IL-4:PE (BD Pharmingen, cl.11B11) and TNFα:APC (eBioscience, cl.MP6-XT22).

### Macrophage infection assay

*Y*. *pseudotuberculosis* YPIII was grown at 25°C overnight in LB broth. A fresh culture was inoculated 1:50 with an overnight culture, grown at 25°C for two hours and then shifted to 37°C for another four hours. Subsequently, bacteria were washed and resuspended in PBS. Immortalized bone marrow derived macrophages were obtained as previously reported [21, 22]. 1 x 10^5^ macrophages were seeded in 96 well plates and infected with 5 x 10^5^ bacteria for one hour. 150 μg gentamicin was added and cells were lysed after one hour. Serial dilutions of bacteria were and plated on LB plates containing 1 μg/ml Triclosan (Calbiochem) to determine the CFUs of invaded bacteria.

### Statistical analysis

Bacterial burden data was analyzed using a Mann-Whitney U test. All other data were analyzed using an unpaired Student’s T-test. Statistical analysis was performed using Graphad Prism (v5.0).

## Results

### Global depletion of NK cells lead to increased bacterial titres of *Y*. *pseudotuberculosis* in the mLNs

It was previously shown by our group that upon infection with *Y*. *pseudotuberculosis* YPIII, NK cell numbers in PPs and mLNs increased while a decrease of NK cells occurred in the spleen and blood [23, 24]. In order to evaluate the overall impact of NK cells during *Y*. *pseudotuberculosis* infections, the anti-NK1.1 antibody mediated NK cell depletion mouse model was employed [20]. In comparison to the alternative anti-Asialo GM1 depletion model, anti-NK1.1 mediated depletion is specific for NK- and NKT-cells [25, 26].

**Fig 1:**
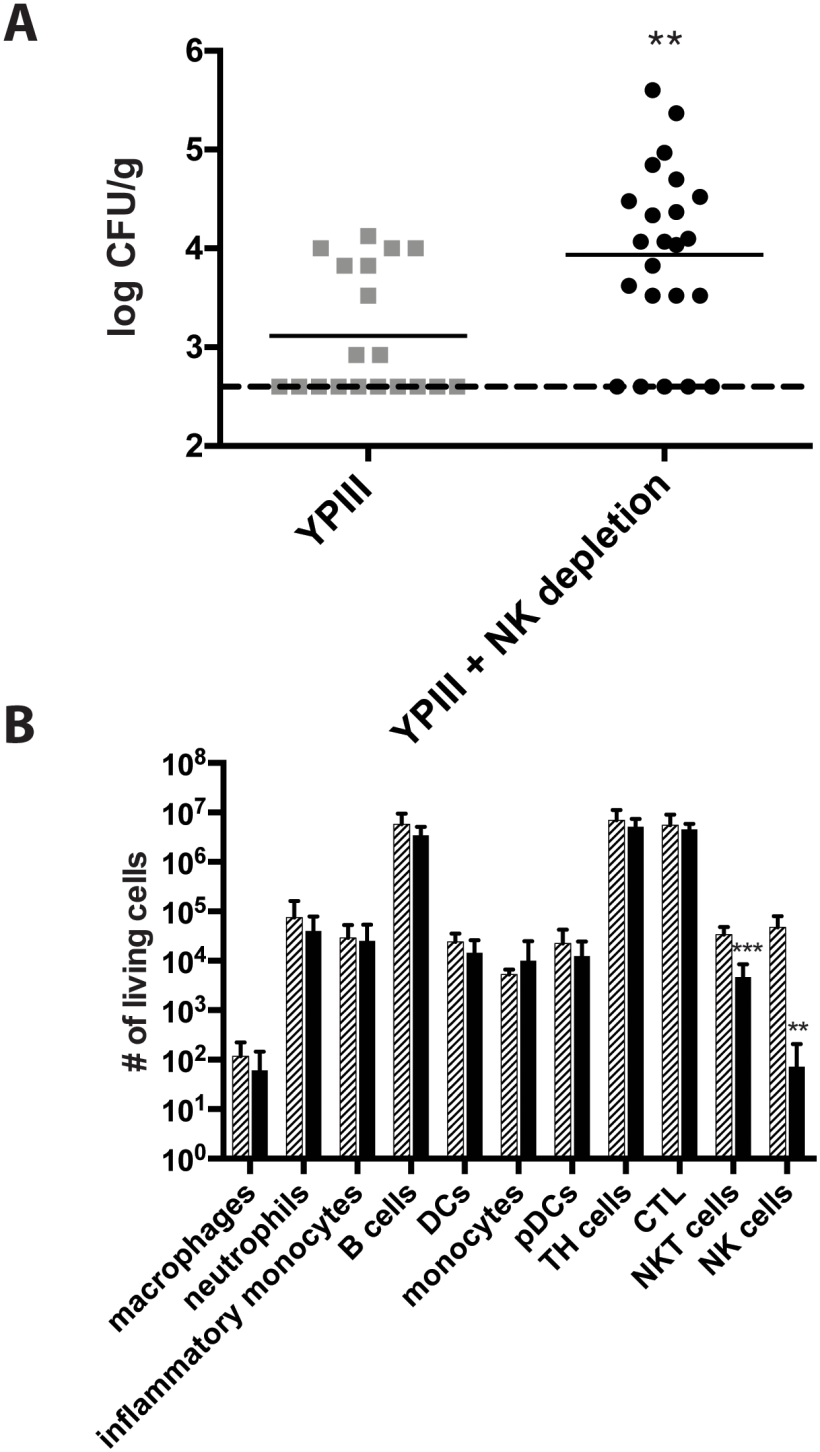
Global depletion of NK cells lead to increased bacterial titres of *Y*. *pseudotuberculosis* in the mLNs. 7-week old female C57BL/6 mice were injected with 100 μg of anti NK1.1 antibody i. p. 24 hours prior to infection. Mice were challenged with 2 x 10^7^ CFU of *Y*. *pseudotuberculosis* strain YPIII. (A) Three days post infection mLNs were excised and homogenates were plated onto selective plates. Data from three independent experiments were pooled. Bacterial loads were compared using a Mann-Whitney U test (**, p < 0.01). (B) Three days post infection mLNs were excised and single cell suspensions were stained with Live/Dead, CD3, CD4, CD8, CD19, NK1.1, CD11b, CD11c, CD49b, F4/80, Ly6C, Ly6G. Living cell numbers of macrophages, neutrophils, inflammatory monocytes, B cells, DCs, monocytes, pDCs, TH cells, CTL, NKT-, and NK cells were assessed. Cross striped bars represent undepleted mice, black bars represent NK depleted mice. Data from three independent experiments were pooled and analyzed with a Student’s t-test (**p < 0.01; ***, p < 0.001). DCs: dendritic cells, TH: T helper cells, CTL: cytotoxic lymphocytes, NKT: natural killer T-cells, NK: natural killer cells.

At first, we compared the organ burden during *Y*. *pseudotuberculosis* infections in NK cell depleted and wild-type mice. On day three post infection PPs, mLNs, spleen and liver of all mice were isolated and the bacterial burden was assessed. The mLNs of NK cell-depleted mice were colonized at significantly higher levels than those of wild-type mice (Fig 1A and S1 Fig). This finding suggests that NK and/or NKT cells might play a protective role against *Y*. *pseudotuberculosis* infection.

It was recently shown that neutrophils play an essential role in *Y*. *pseudotuberculosis* clearance [26]. Moreover, CD8^+^ T-cells were demonstrated to participate in bacterial clearance at later stages of infection [27]. Hence, we investigated whether the compartment of innate immune cells in the mLNs changed during infection in both NK depleted and undepleted mice (gating strategy: S2 and S3 Figs). Upon infection, the number of neutrophils and inflammatory monocytes increased in the mLNs independently of precedent NK depletion (Fig 1B). When comparing the cell composition in infected mLNs of NK-depleted and undepleted mice, no difference could be observed in any of the tested cell types except the anticipated decrease in NK and to a smaller extent of NKT cells (Fig 1B). In addition, we did not observe any changes in cell distribution comparing uninfected untreated and NK cell depleted mice (S4 Fig). These results suggest that the observed differences in the bacterial load does not depend on differences in the recruitment of phagocytes, but are directly linked to the absence of NK and/or NKT cells.

### CD11b^+^ CD27^+^ NK cell frequency increases in the mLNs during a *Y*. *pseudotuberculosis* infection

**Fig 2:**
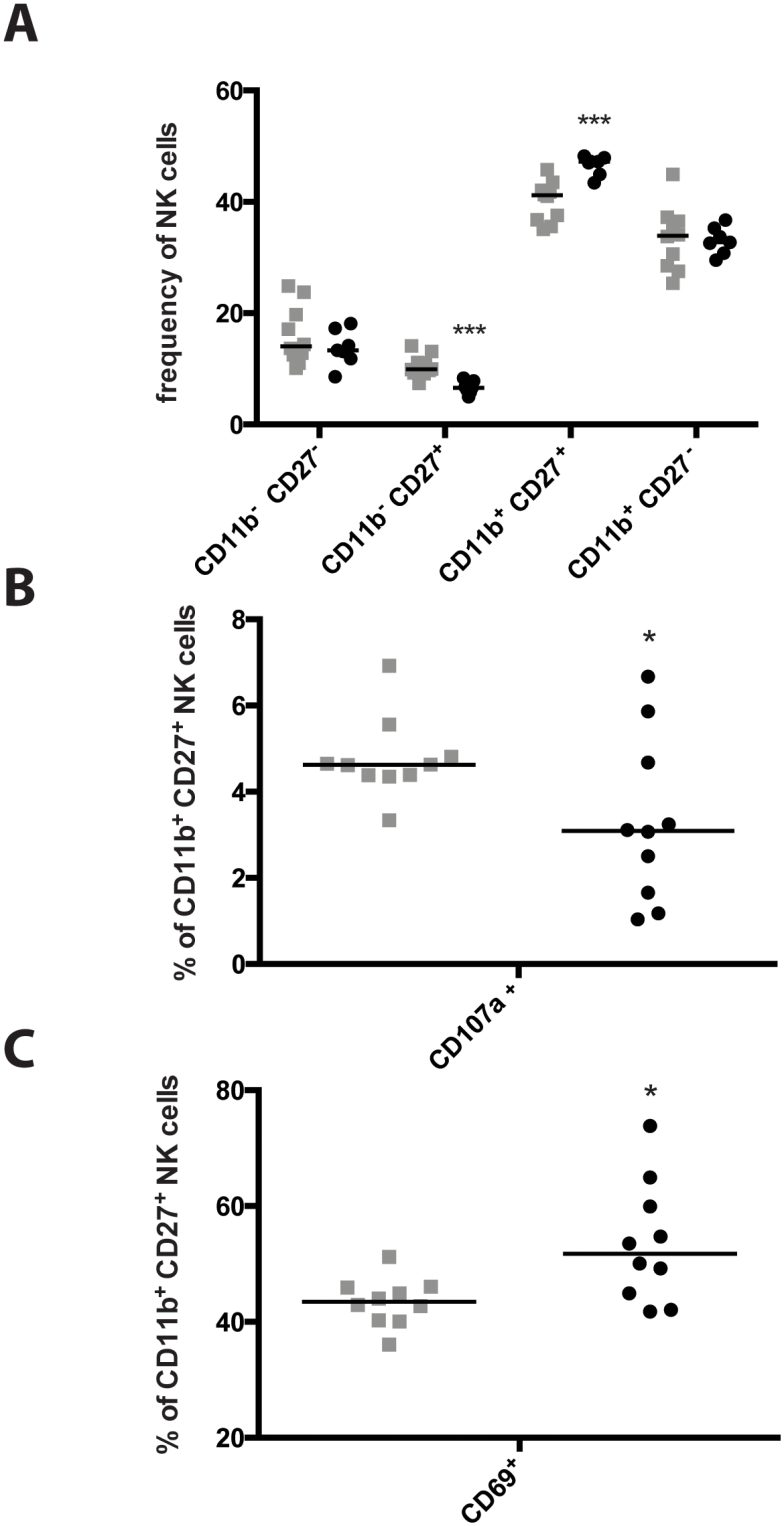
CD11b^+^, CD27^+^ NK cell frequency increases in the mLN during infection with *Y*. *pseudotuberculosis* YPIII. (A) Mice were infected with 2 x 10^7^ CFU of *Y*. *pseudotuberculosis* strain YPIII. Three days post infection mLN were excised and NK cell population distribution was analyzed by flow cytometry. To do so, cells were stained with Live/Dead, CD3, NK1.1, CD11b, CD27, CD69 and CD107a. Grey filled squares represent uninfected mice, black filled circles illustrate *Y*. *pseudotuberculosis* infected mice. CD11b^+^, CD27^+^ NK cells from the mLN were further analyzed for their expression profiles of the surface markers CD107a (B) and CD69 (C). Grey filled squares represent uninfected mice, while black filled circles represent YPIII infected mice. Data from three independent experiments were pooled and analyzed with a Student’s t-test (*, p < 0.05; ***, p<0.001).

During their development, NK cells exhibit different functions, which are associated to the expression of specific cell surface markers [28]. However, whether individual subsets of NK cells have an influence on bacterial infections or not is largely unknown. To address this question we investigated if specific NK cell populations occur in the mLNs of *Y*. *pseudotuberculosis*-infected mice. Two markers were used for assessing NK cell subset differentiation in this study—CD11b and CD27 (S5 Fig). They represent distinct stages of NK cells from different tissues with unique functional and phenotypic attributes [29]. Three days post infection we observed an increased frequency of CD11b^+^ CD27^+^ NK cells and a lower frequency of CD11b^-^ CD27^+^ NK cells in the mLNs (Fig 2A), indicating a shift in the NK cell subsets during the early stages of the infection. We further tested the expression of the activation marker CD69 and the degranulation marker CD107a. The analysis revealed a small reduction of CD107a expression in CD11b^+^ CD27^+^ NK cells (Fig 2B), suggesting that the cytotoxic activity of the NK cell subset is slightly decreased. However, a substantial increase in the amount of CD69^+^ cells was observed in the CD11b^+^ CD27^+^ NK cell population, indicating a higher activation status of this subset (Fig 2C). These data show that NK cells in the mLNs are activated upon infection with *Y*. *pseudotuberculosis* and, based on the expression of CD69, are likely to produce proinflammatory cytokines in response to the infection (Fig 2B).

### NK cells in the mLNs are essential for IFNγ production and induce TNFα secretion

**Fig 3:**
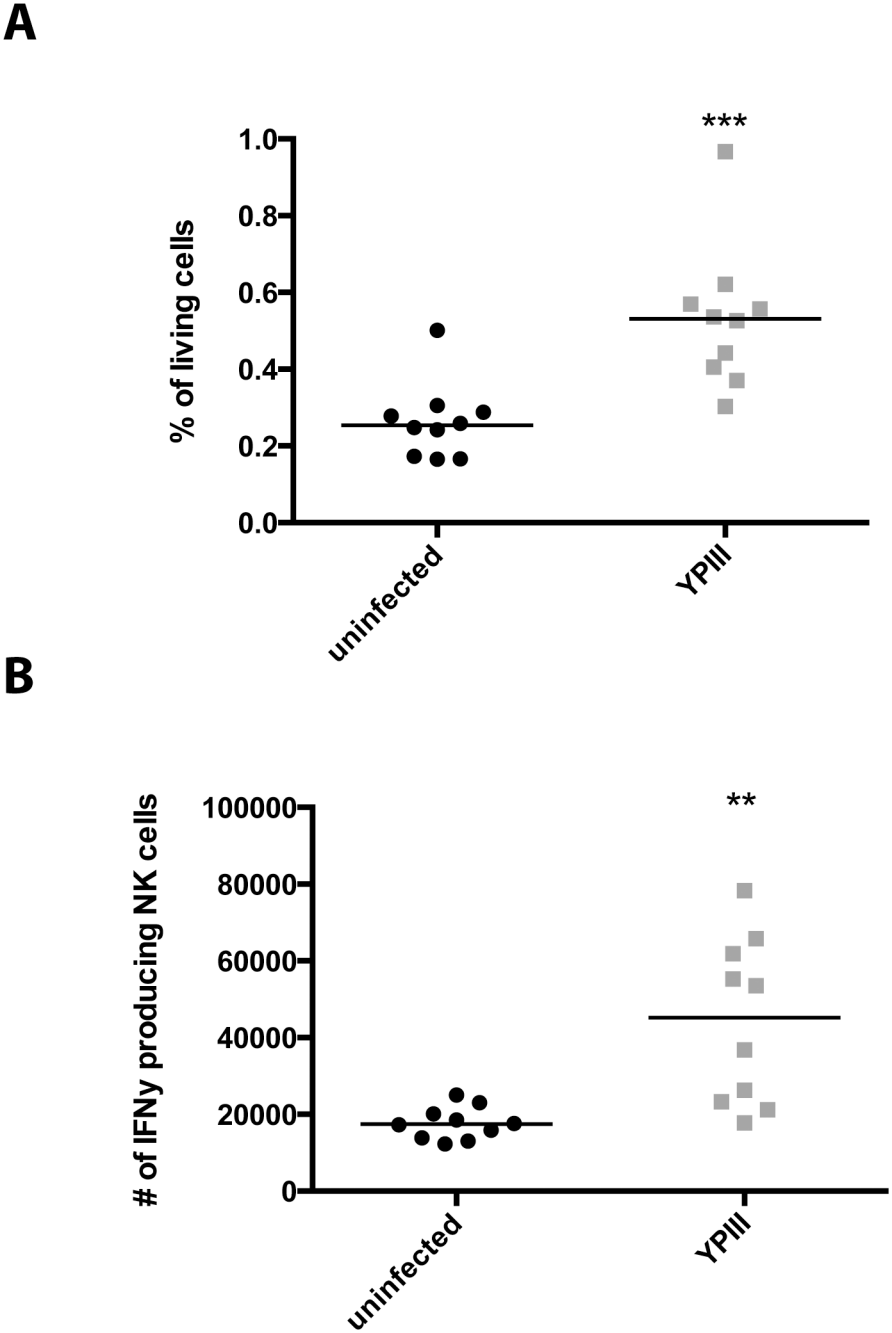
Infection with *Y*. *pseudotuberculosis* results in an increased frequency and numbers of IFNγ producing NK cells. Mice were infected with 2 x 10^7^ CFUs of *Y*. *pseudotuberculosis* strain YPIII. Three days post infection mLN were excised, single cell suspensions were prepared and used for extracellular (CD3, NK1.1) and intracellular staining (IFNγ). Data from three independent experiments were pooled and analyzed with a Student’s t-test, and the frequency (A) and total number (B) are given (**, p < 0.01; ***, p < 0.001).

Activation of NK cells has been reported to be mainly associated with an increased production of IFNγ. We therefore investigated whether *Y*. *pseudotuberculosis* is able to stimulate IFNγ production during the early stages of the *Y*. *pseudotuberculosis* infection by intracellular staining using anti-IFNγ antibodies and flow cytometry (S6 Fig). Our experiments revealed a significant increase in the frequency (Fig 3A) and total number of IFNγ-producing NK cells in the mLNs (Fig 3B). In order to evaluate the role of NKT cells in the present model, we performed cytokine staining (IFNγ, TNFα and IL-4) three days post infection. No significant differences were observed comparing uninfected and YPIII infected samples (S7 Fig). These results indicate that the observed phenotype is very likely attributable to NK cells rather than NKT cells.

**Fig 4:**
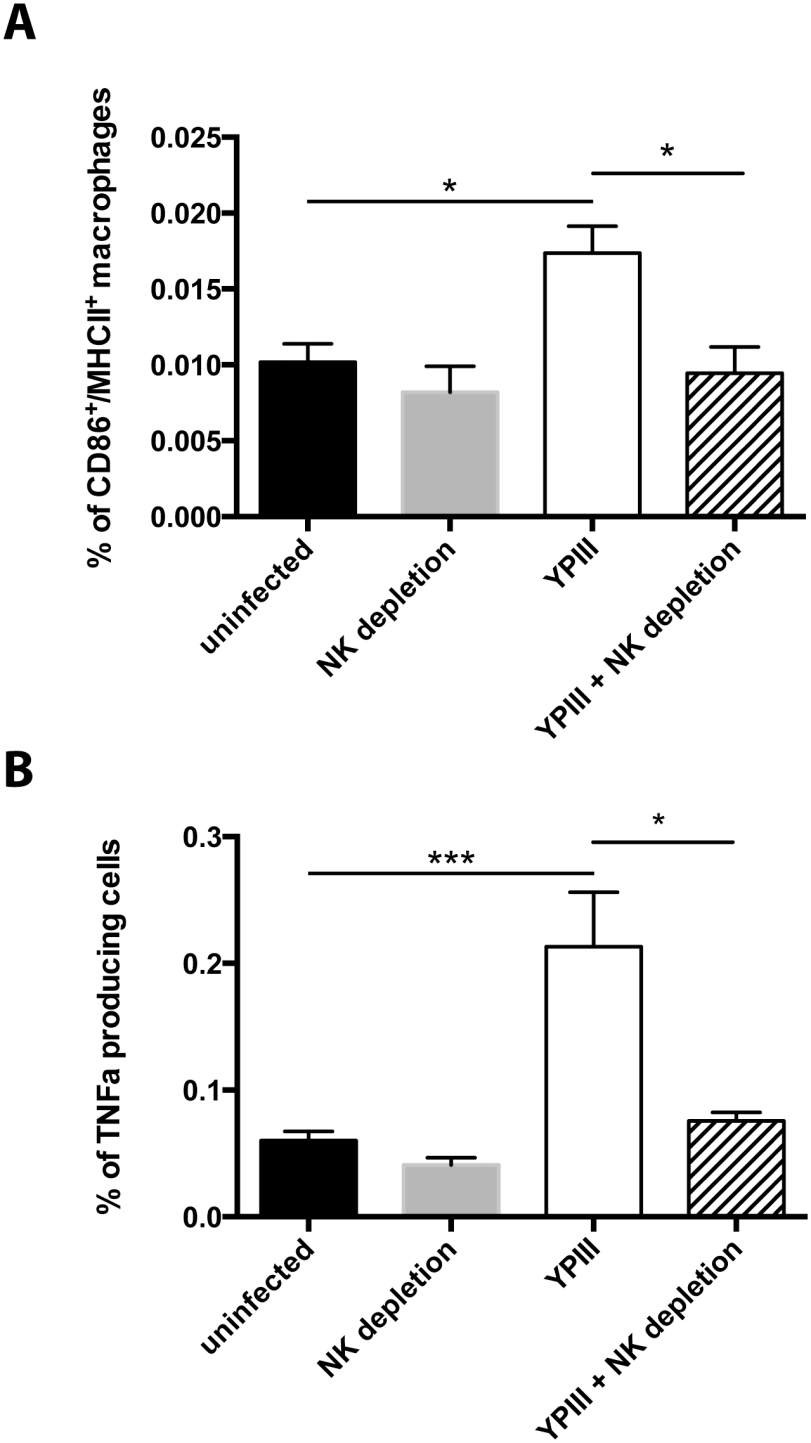
TNFα production by macrophages in the mLN is dependent on the presence of NK cells. 24 hours prior to infection mice were injected with 100 μg of anti NK1.1 antibody i.p. mice were infected with 2 x 10^7^ CFUs of *Y*. *pseudotuberculosis* YPIII. Three days post infection mLNs were isolated, single cell suspension were prepared and used for extracellular (CD3, CD11b, CD19, CD86, CD49b, MHCII, F4/80) and intracellular staining (TNFα). (A) Depicted are frequencies of CD86^+^/MHCII^+^ macrophages among living cells. CD11b^+^ F4/80^+^ cells were defined as macrophages. Subset analysis with CD86 / MHCII surface markers was performed. (B) Single cells were analyzed for their expression of TNFα and subsequently the expression of either CD3/CD19/CD49b or F4/80 to determine their cell type. Depicted are F4/80^+^ macrophages producing TNFα. Data from two independent experiments were pooled and analyzed with a Student’s t-test (*, p<0.05; ***, p<0.001).

IFNγ is a proinflammatory cytokine whose functions range from the induction of other proinflammatory cytokines (e.g. IL-2) to macrophage activation [30]. Macrophage activation was assessed via staining with CD86/MHCII as classical macrophage activation markers (Fig 4A). We found significantly more CD86^+^ MHCII^+^ macrophages in the mLNs of infected samples compared to uninfected mice. This effect was only seen in NK cell harboring mice supporting our hypothesis of NK cell produced IFNγ-mediated macrophage activation. Classical macrophage activation can lead to production of TNFα. TNFα mediates both local and systemic inflammatory responses and influences development of pathological reactions e.g. when challenged with *Y*. *enterocolitica* [31, 32]. We employed intracellular staining using an anti-TNFα antibody to address the dynamics of TNFα synthesis via flow cytometry. Our data show a significant increase in the frequency of TNFα-producing macrophages upon infection with *Y*. *pseudotuberculosis* compared to uninfected mice (Fig 4B). Additionally, we investigated the dependency of TNFα production on the presence of NK cells. Our results clearly demonstrate that TNFα accumulation in the mLNs is abolished upon NK cell depletion, showing that production of this pleiotropic proinflammatory cytokine is solely dependent on the presence of NK cells (Fig 4B).

**Fig 5:**
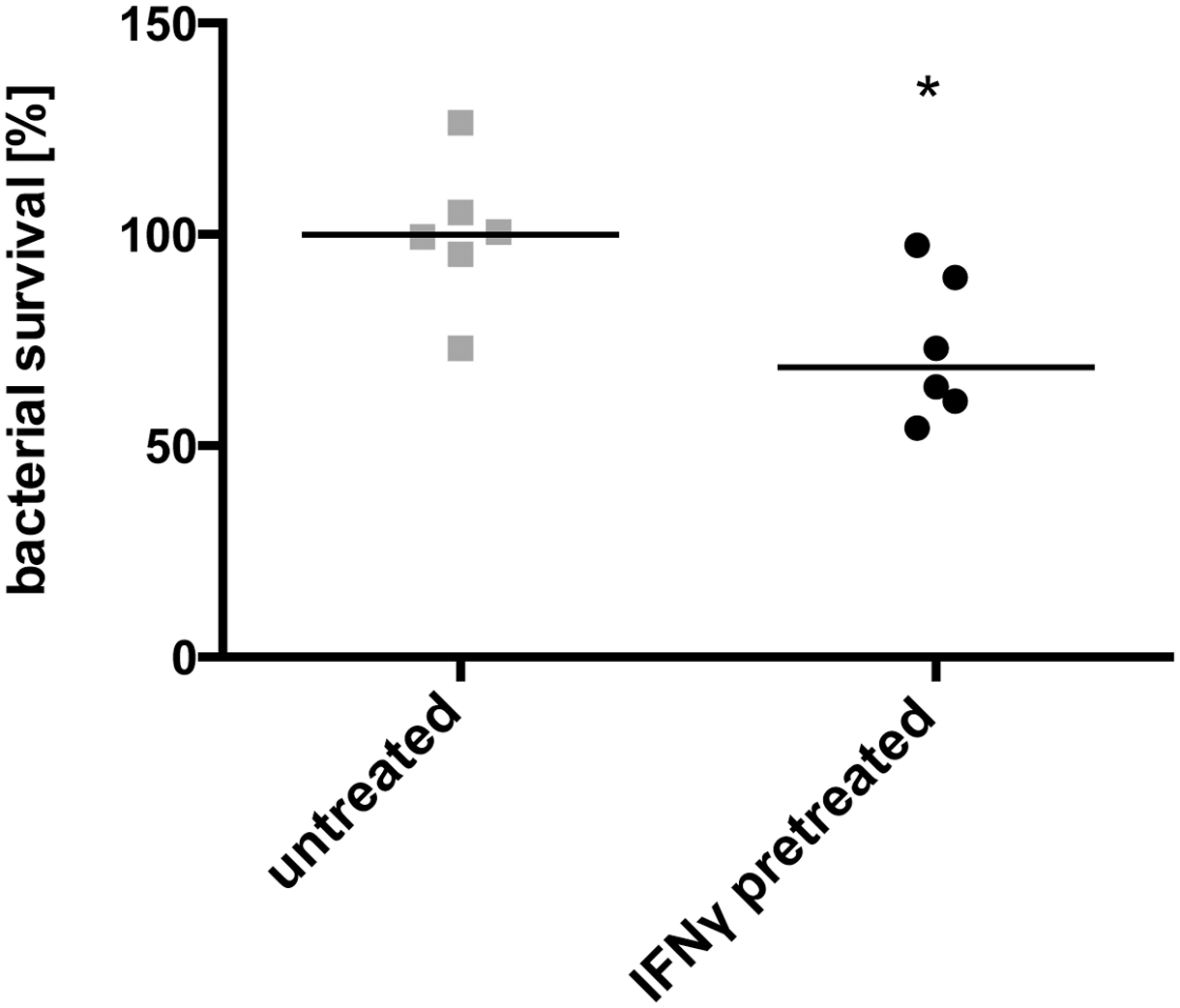
*Y*. *pseudotuberculosis* YPIII killing by macrophages is increased after IFNγ prestimulation. 1 x 10^6^ macrophages were infected with MOI 10 of YPIII, 1 hour post infection gentamycin treatment killed extracellular bacteria and after additional 60 minutes of incubation cells were lysed and bacteria were plated onto selective plates. The mean of the survival of untreated macrophage was used as baseline. Data from two independent experiments were pooled and analyzed with a Student’s t-test (*, p<0.05).

Since NK cell-derived IFNγ can activate macrophages, we then tested whether macrophage-mediated killing of *Y*. *pseudotuberculosis* is influenced by a pretreatment of immortalized bone marrow derived macrophages with IFNγ. Previous studies demonstrated that phagosome-lysosome fusion and subsequent bacterial killing of *Yersinia* in murine macrophages does not only depend on the state of macrophage activation, but also on the activity of the T3SS/Yop machinery [9, 33]. *Y*. *pseudotuberculosis* that actively translocates the Yop effectors activates multiple cytosolic signaling pathways to protect the pathogen from autophagy-dependent killing. For this reason, we studied influence of IFNγ on macrophage-mediated killing with bacteria grown under T3SS/Yop-inducing conditions. As shown in Fig 5, our experiments revealed that prestimulation of the macrophages with IFNγ improved killing of *Y*. *pseudotuberculosis*.

## Discussion

NK cells constitute important sentinels of the innate immune system and act as initial responder to a number of bacterial pathogens [12]. They become activated by cytokines secreted from stimulated DCs and macrophages or through interaction with infected cells. In response, NK cells discharge exocytotic granules, e.g. perforin and granzymes to induce cell lysis or they secrete cytokines to trigger an effective immune response [12, 13].

It has long been speculated that NK cells play a role in the early phase of the host defense against *Yersinia* infections. This assumption was based on an observation that NK cells from BALB/c mice, which are susceptible to *Y*. *enterocolitica*, react with a rather weak induction of IFNγ in contrast to the more resistant C57BL/6 mice with a strong IFNγ response [34]. It was further shown that IL-12 is essential for resistance against *Y*. *enterocolitica* by triggering IFNγ production in NK and T-cells [18]. In this study, we initiated depletion of NK cells with anti-NK1.1 antibodies to study the influence of NK cells in clearing *Y*. *pseudotuberculosis* infections. Our infection experiments revealed that NK cell depletion increased the bacterial burdens in the mLNs during the early stages of the infection and provide the first direct evidence for a role of NK cells in the control of *Y*. *pseudotuberculosis*. Depletion of NK cells was also found to influence the bacterial titres during a *Y*. *enterocolitica* infection. However, in this case a significantly lower number of bacteria was detectable only in the spleen [18]. Whether this is based on differences between the species is unclear, since the NK cell-depleting anti-asialo GM1 antibody was used in this study, which was later shown to have also a drastic effect on the activity of cytotoxic T lymphocytes and basophils [25, 26].

Implication of NK cells in antibacterial immunity is now widely accepted, but the influence of these leukocytes seems to depend on the pathogen and the circumstances of the infection [14, 35]. NK cells were initially found to control intracellular pathogens such as *Shigella flexneri*, *Legionella pneumophila*, and *Mycobacterium avium* by lysis of pathogen-infected phagocytes [14]. More recently, NK cells have also been shown to be key players in early immunity against extracellular pathogens. For instance, NK cells were recruited to the colon, mLNs and spleen during a *Citrobacter rodentium* infection, where they promote clearance of the pathogen [36, 37].

NK cells are mainly required for phagocyte activation and maturation, antigen presentation and priming of T cell responses [13]. We specifically found CD11b^+^ CD27^+^ NK cells to be enriched upon a *Y*. *pseudotuberculosis* infection. CD11b^+^ CD27^+^ NK cells are mature NK cells, which were shown to have the greatest ability to secrete cytokines (e.g. IFNγ) after stimulation [29]. In accordance, a significantly higher frequency of CD69^+^ cells was found among the CD11b^+^ CD27^+^ population of NK cells in the mLNs of infected mice. Moreover, a considerable increase of NK cell-triggered IFNγ production was detectable during the early stages of a *Y*. *pseudotuberculosis* infection and stimulation of macrophages with IFNγ was shown to decrease survival of *Y*. *pseudotuberculosis* when incubated with professional phagocytes.

A protective role of IFNγ has also previously been reported for *Y*. *enterocolitica*. Administration of IFNγ antibodies rendered BALB/c mice highly susceptible towards *Y*. *enterocolitica* infections [18]. Furthermore, a significant increase in IFNγ production was observed in the spleen during *Y*. *enterocolitica* infection [15]. Most of the IFNγ is produced by CD4^+^ T-cells at later stages of the infection. NK cells are responsible for the production of the major portion of IFNγ during early phases of the infection. However, IFNγ derived from these immune cells was not essential and could be compensated by IFNγ from CD4^+^ T-cells [15]. Moreover, IFNγ was reported to be critical for CD8 T-cell mediated protection against pulmonary *Y*. *pestis* infections [38].

Similar to *Yersinia*, NK cell-mediated production of IFNγ was also observed during infections with other enteric pathogens, e.g. *C*. *rodentium*, *S*. *enterica* serovar Typhimurium and *L*. *monocytogenes*, but the outcome varied significantly among the bacterial infections. For instance, it has been reported that similar to *Yersinia*, IFNγ promotes phagocyte-mediated killing of *Salmonella* [39]. Other reports further documented that NK cell-mediated IFNγ secretion contributes to the *in vivo* control of *Salmonella* [40–42]. In the case of a *C*. *rodentium* infection, loss of NK cells resulted in lower colonic IFNγ and a delay in homing of IFNγ^+^ CD4^+^ T cells to the gut. The decrease of IFNγ levels did not reduce, but increased levels of pathology, suggesting that IFNγ is important to limit intestinal damage [36]. NK cells respond to sublethal doses of *L*. *monocytogenes* during the infection of rats by an induction of IFNγ in the spleen, whereas a lethal dosis of the same pathogen leads to excessive IFNγ production in mice, which impairs granulocyte migration to the spleen and reduces *Listeria* clearance [20, 43].

Considering the role of NK cell-derived IFNγ in the combat of infections, it is not surprising that some bacteria, including *yersiniae*, have evolved defense strategies to inhibit NK cell functions [23, 41, 44–48]. Studies addressing whether *yersiniae* are able to target host NK cells revealed that *Y*. *pestis*, *Y*. *enterocolitica* and *Y*. *pseudotuberculosis* interact with NK cells and manipulate their function through translocated Yop effectors [23, 44, 48]. However, no common picture has evolved on how they influence NK cells to inhibit immune responses. *Y*. *enterocolitica* O:8 strain WA interacts with NK cells *in vitro* and inhibits NK cytotoxicity as well as the expression of IFNγ and other IL12-/IL18-induced factors through the T3SS effector YopP [44]. Inactivation of NK cell function seems to be a consequence of YopP-induced NK cell apoptosis, and indirect effects reducing cytokine production by targeted antigen-presenting cells and/or accessory NK cell activating cells [44]. For *Y*. *pestis* KIM5 it was shown that YopM counteracts innate immune responses by causing a global decrease in NK cells during the early stages of the infection [23]. A reduction of NK cells was also observed in the spleen of mice infected with *Y*. *pseudotuberculosis* IP2666. However, NK cell depletion did not occur in a YopM-dependent manner and appeared to be caused by higher bacterial loads obtained with wildtype- compared to *yopM* mutant-infected mice [48].

We recently showed that NK cells in the mLN and spleen are also subject to Yop effector translocation during a *Y*. *pseudotuberculosis* YPIII infection [49]. The number of NK cells was substantially decreased in the spleen, but increased in the mLNs and PPs of infected mice at day three post infection [24]. Furthermore, we found that an elimination of the T3SS/Yop machinery did not result in a decrease of the bacterial burden of the mLNs, but in all other infected tissues [50]. This demonstrated the T3SS/Yop machinery is not essential for bacterial survival in the mLNs where the presence of NK cells is particularly important to reduce the bacterial numbers. Thus, in the mLNs, NK cell function does not seem to be affected by Yop effectors.

In summary, our work suggests a protective function of NK cells in modulating IFNγ levels in response to an oral *Y*. *pseudotuberculosis* infection, leading to clearance of the bacteria from the mLNs during the early phases of infection. Although we cannot exclude NK cell-mediated killing of *Y*. *pseudotuberculosis* in our *in vivo* models, cytotoxic responses seem less important, since only the subpopulation expressing the activation marker CD69 expanded during the infection. Based on these data we favor a model in which activated NK cells respond indirectly to *Yersinia*-infected host cells by production of proinflammatory cytokines.

## Supporting Information

S1 Fig NK cells protect against *Y*. *pseudotuberculosis* infections in the mLN three days post infection. 7-week old female C57BL/6 mice were injected with 100 ug of anti NK1.1 antibody i. p. 24 hours prior to infection. Mice were challenged with 2 x 10^7^ CFU of *Y*. *pseudotuberculosis* strain YPIII. Three days post infection PPs, mLNs, liver and spleen were excised and homogenates were plated onto selective plates. Data from three independent experiments were pooled. Bacterial loads were compared using a Mann-Whitney U test (**, p < 0.01).
(TIF)

S2 Fig Gating strategy for adaptive cell compartments + NK/NKT cells. Three days post infection mLNs were excised and single cell suspensions were stained with Live/Dead (LD), CD3, CD4, CD8, CD19, NK1.1. Living cell numbers of B cells (CD19^+^ CD3^-^ NK1.1^-^) CD4^+^ T-cells (CD19^-^ CD3^+^ NK1.1^-^ CD4^+^), CD8^+^ T-cells (CD19^-^ CD3^+^ NK1.1^-^ CD8^+^), NKT-cells (CD19^-^ CD3^+^ NK1.1^+^), and NK cells (CD19^-^ CD3^+^ NK1.1^+^) were analyzed.
(TIF)

S3 Fig Gating strategy for innate cell compartments. Three days post infection mLNs were excised and single cell suspensions were stained with Live/Dead (LD), CD3, CD49b, CD19, CD11b, CD11c, F4/80, Ly6C and Ly6G. Following exclusion of B-/T-/NK cells (CD19^+^ CD3^+^ CD49b^+^), macrophages (CD19^-^ CD3^-^ CD49b^-^ F4/80^hi^), neutrophils (CD19^-^ CD3^-^ CD49b^-^ F4/80^low/int^ Ly6G^+^ CD11b^+^) dendritic cells (CD19^-^ CD3^-^ CD49b^-^ F4/80^low/int^ Ly6G^-^ Ly6C^low^ CD11c^+^) pDCs (CD19^-^ CD3^-^ CD49b^-^ F4/80^low/int^ Ly6G^-^ CD11c^-^ CD11b^-^ Ly6C^+^) monocytes (CD19^-^ CD3^-^ CD49b^-^ F4/80^low/int^ Ly6G^-^ CD11c^-^ Ly6C^-^ CD11b^+^) and inflammatory monocytes (CD19^-^ CD3^-^ CD49b^-^ F4/80^low/int^ Ly6G^-^ CD11c^-^ Ly6C^+^ CD11b^+^) were analyzed.
(TIF)

S4 Fig Depletion of NK and NKT cells with the NK1.1 antibody. 7-week old female C57BL/6 mice were injected with 100 ug of anti NK1.1 antibody i. p. Three days post infection mLNs were excised and single cell suspensions were stained with Live/Dead (LD), CD3, CD4, CD8, CD19, NK1.1, CD11b, CD11c, CD49b, F4/80, Ly6C, Ly6G. Living cell numbers of dendritic cells (DCs), neutrophils, macrophages, monocytes, inflammatory monocytes, pDCs, T helper cells (T_H_ cells) cytotoxic T lymphocytes (CTL), NK cells, NKT cells and B cells were assessed. Black bars represent undepleted mice, white bars represent NK depleted mice. Data from three independent experiments were pooled and analyzed with a Students t-test (*, p < 0.05).
(TIF)

S5 Fig Gating strategy for NK cell subset differentiation. Three days post infection mLNs were isolated and stained with the following markers to differentiate NK cell subsets: Live/Dead (LD), CD3, NK1.1, CD11b, CD27, CD69, CD107a. After gating for living cells and doublet exclusion NK cells (CD3^-^ NK1.1^+^) were further analyzed for their expression of CD11b and CD27. Subsets were defined a seither CD11b^+/-^ and/or CD27^+/-^. CD11b^+^ CD27^+^ cells underwent additional analysis of their CD69 and CD107a expression.
(TIF)

S6 Fig Gating strategy for cytokine production analysis. Three days post infection mLNs were isolated and stained with the following markers to differentiate cytokine producers: Live/Dead (LD), CD3, NK1.1, IFNγ and TNFα. Cells were first analyzed for their expression of the respective cytokine and afterwards the producing cells were associated with either CD3 for T-cells, NK1.1 for NK cells or expression of neither (of non T-cell, non NK cell origin)/ both (NKT cells).
(TIF)

S7 Fig Influence of NKT cell produced cytokines. Three days post infection mLNs were isolated and stained with the following markers to differentiate cytokine producers: Live/Dead (LD), CD3, NK1.1, IFNγ, IL-4 and TNFα. Cells were analyzed for their expression of NKT surface markers (CD3^+^ NK1.1^+^). Subsequently, expression of the respective cytokines was investigated.
(TIF)
