# Supplementary figures and images for: Natural Killer Cells Mediate Protection against Yersinia pseudotuberculosis in the Mesenteric Lymph Nodes
Source: PLoS One. 2015 Aug 21;10(8):e0136290. doi: 10.1371/journal.pone.0136290 (PMC4546584; doi:10.1371/journal.pone.0136290)

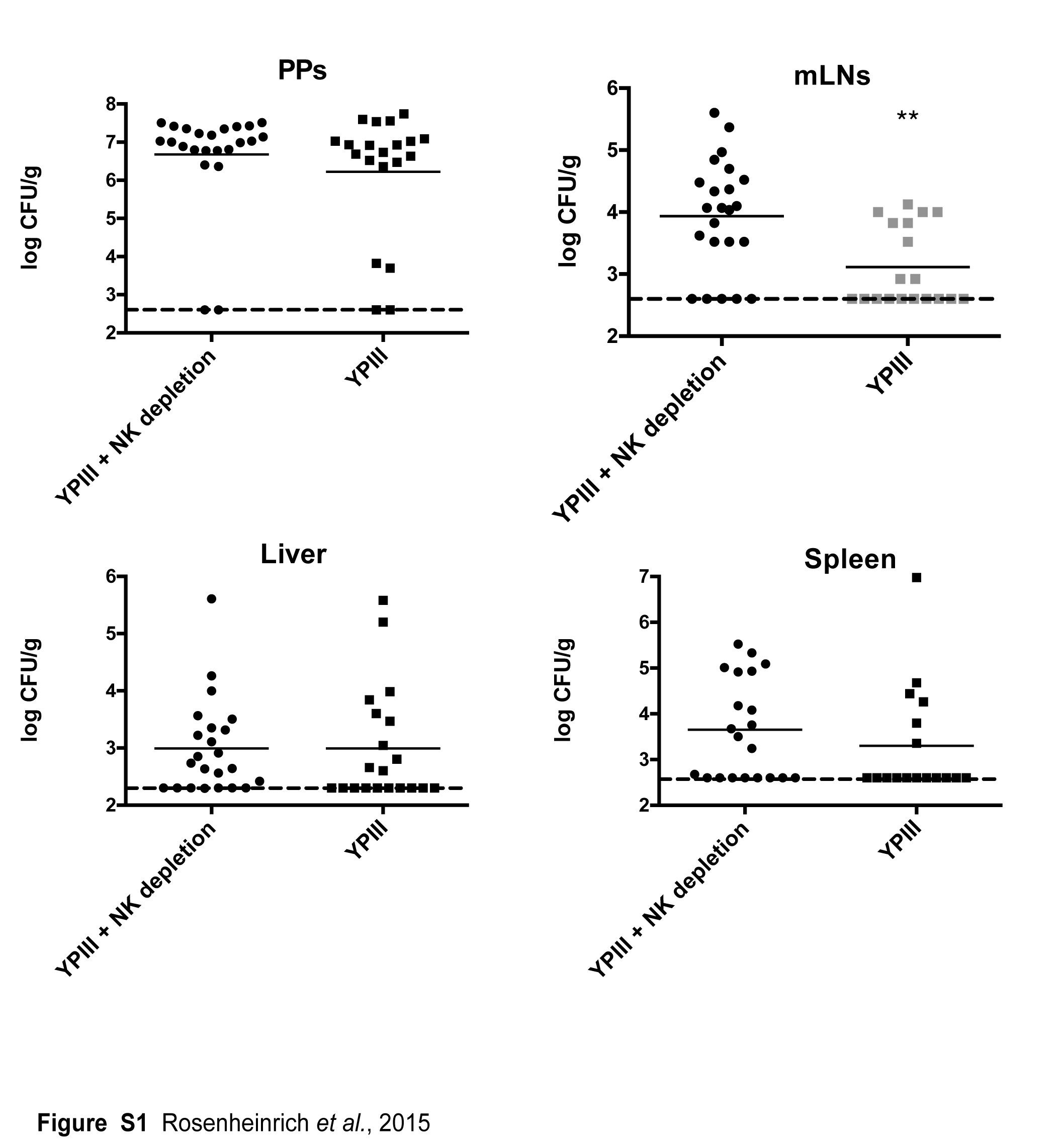

Supplement: S1 Fig — 7-week old female C57BL/6 mice were injected with 100 ug of anti NK1.1 antibody i. p. 24 hours prior to infection. Mice were challenged with 2 x 10^7^ CFU of Y. pseudotuberculosis strain YPIII. Three days post infection PPs, mLNs, liver and spleen were excised and homogenates were plated onto selective plates. Data from three independent experiments were pooled. Bacterial loads were compared using a Mann-Whitney U test (**, p < 0.01). (TIF) [file pone.0136290.s001.tif]

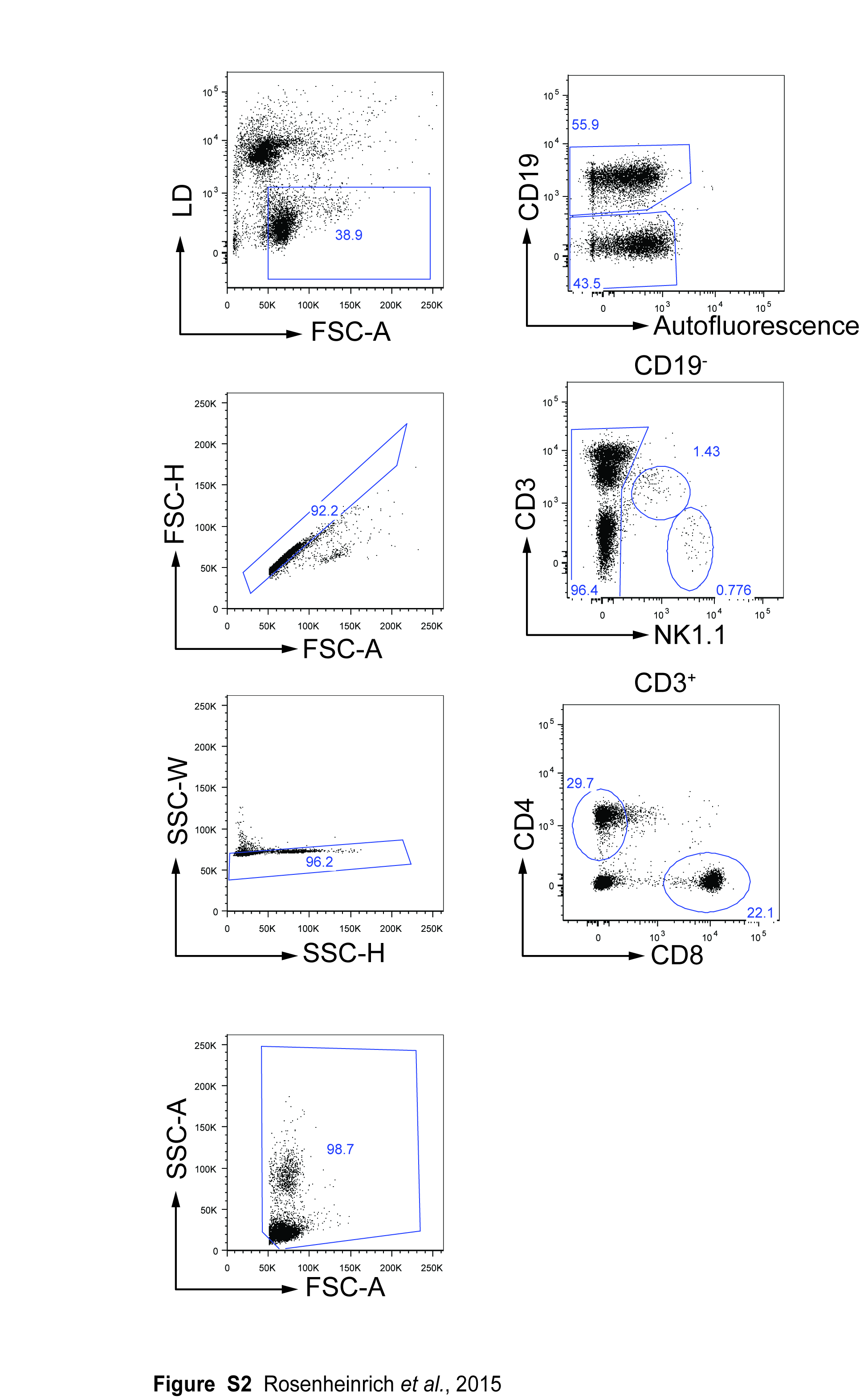

Supplement: S2 Fig — Three days post infection mLNs were excised and single cell suspensions were stained with Live/Dead (LD), CD3, CD4, CD8, CD19, NK1.1. Living cell numbers of B cells (CD19+ CD3- NK1.1-) CD4+ T-cells (CD19- CD3+ NK1.1- CD4+), CD8+ T-cells (CD19- CD3+ NK1.1- CD8+), NKT-cells (CD19- CD3+ NK1.1+), and NK cells (CD19- CD3+ NK1.1+) were analyzed. (TIF) [file pone.0136290.s002.tif]

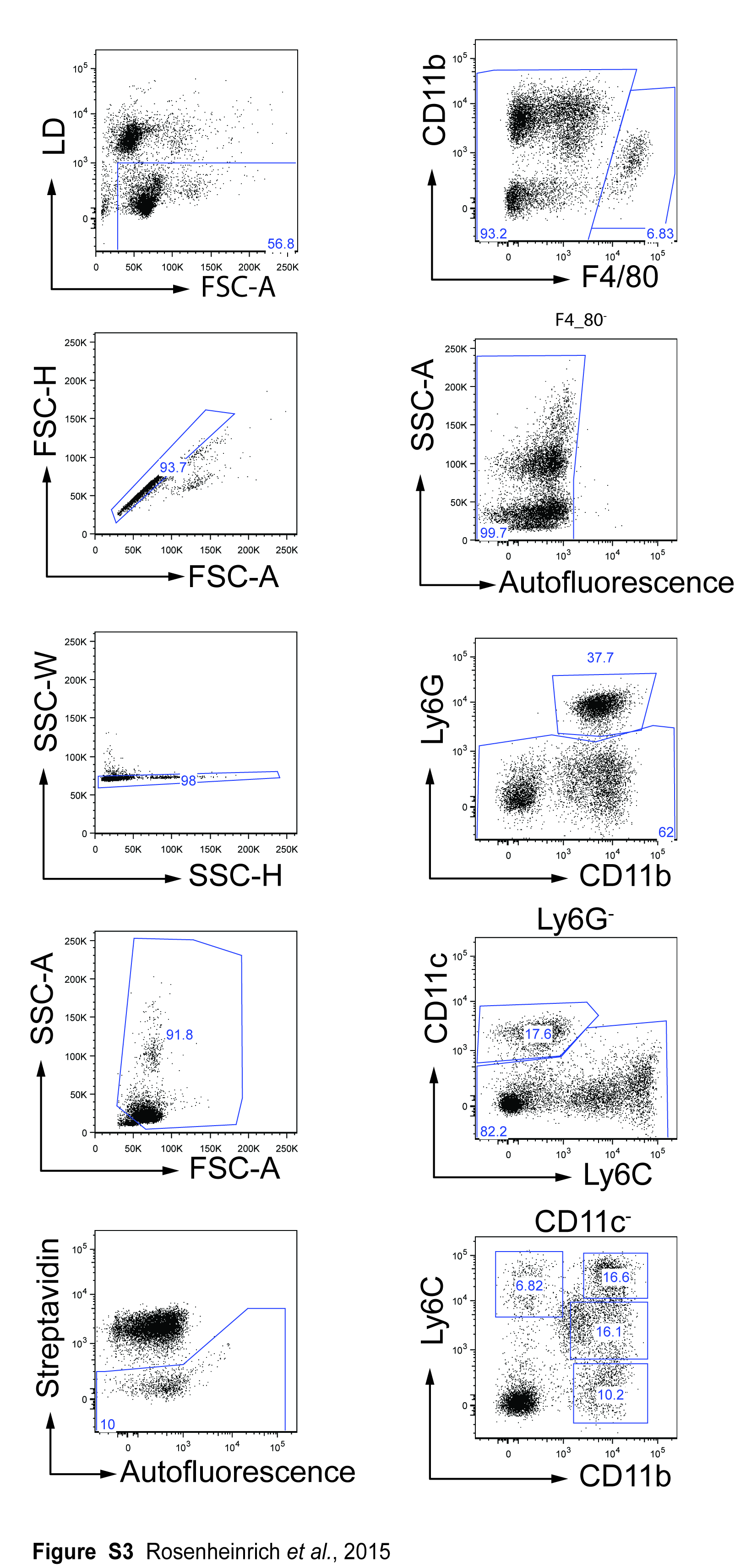

Supplement: S3 Fig — Three days post infection mLNs were excised and single cell suspensions were stained with Live/Dead (LD), CD3, CD49b, CD19, CD11b, CD11c, F4/80, Ly6C and Ly6G. Following exclusion of B-/T-/NK cells (CD19+ CD3+ CD49b+), macrophages (CD19- CD3- CD49b- F4/80hi), neutrophils (CD19- CD3- CD49b- F4/80low/int Ly6G+ CD11b+) dendritic cells (CD19- CD3- CD49b- F4/80low/int Ly6G- Ly6Clow CD11c+) pDCs (CD19- CD3- CD49b- F4/80low/int Ly6G- CD11c- CD11b- Ly6C+) monocytes (CD19- CD3- CD49b- F4/80low/int Ly6G- CD11c- Ly6C- CD11b+) and inflammatory monocytes (CD19- CD3- CD49b- F4/80low/int Ly6G- CD11c- Ly6C+ CD11b+) were analyzed. (TIF) [file pone.0136290.s003.tif]

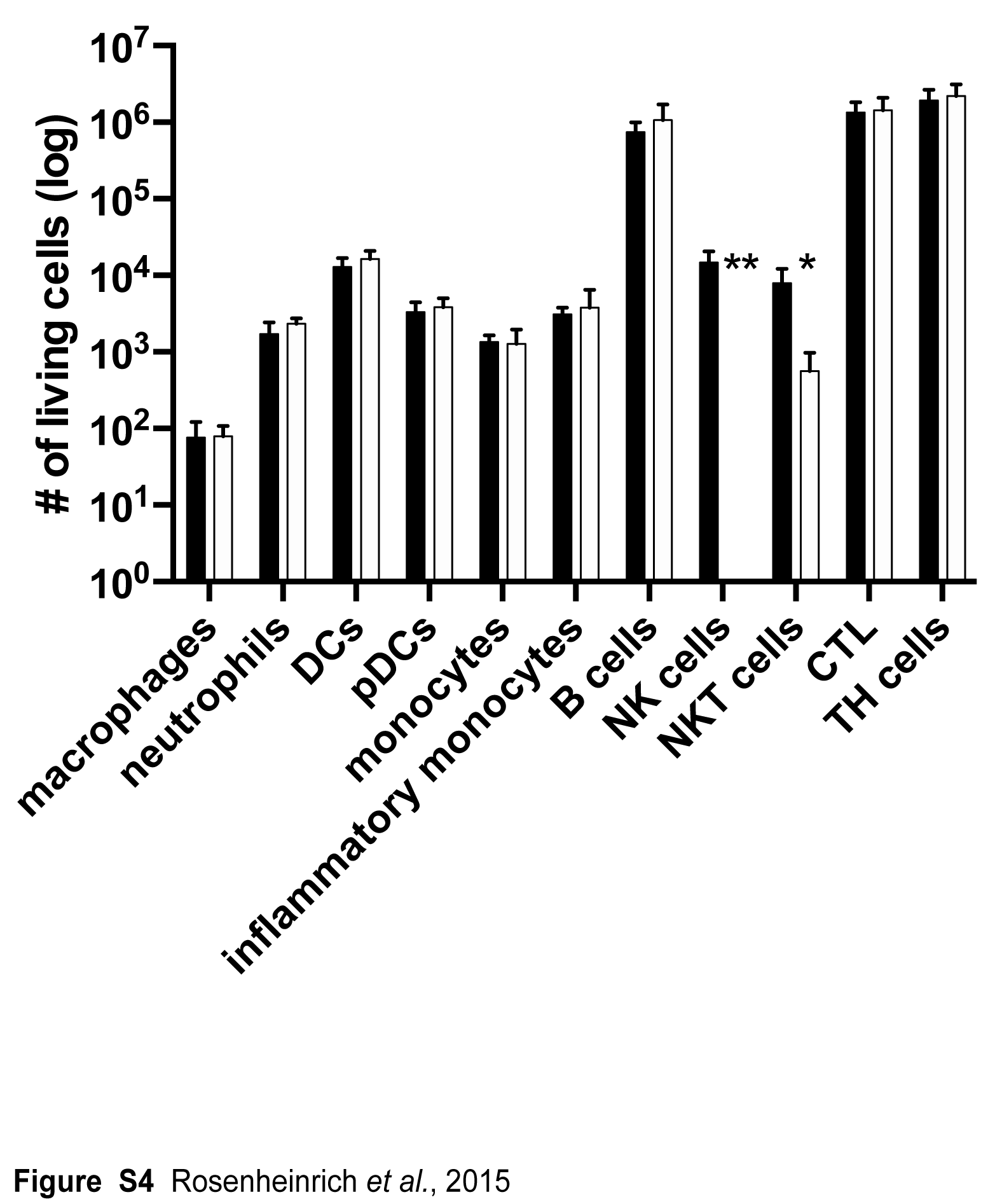

Supplement: S4 Fig — 7-week old female C57BL/6 mice were injected with 100 ug of anti NK1.1 antibody i. p. Three days post infection mLNs were excised and single cell suspensions were stained with Live/Dead (LD), CD3, CD4, CD8, CD19, NK1.1, CD11b, CD11c, CD49b, F4/80, Ly6C, Ly6G. Living cell numbers of dendritic cells (DCs), neutrophils, macrophages, monocytes, inflammatory monocytes, pDCs, T helper cells (TH cells) cytotoxic T lymphocytes (CTL), NK cells, NKT cells and B cells were assessed. Black bars represent undepleted mice, white bars represent NK depleted mice. Data from three independent experiments were pooled and analyzed with a Students t-test (*, p < 0.05). (TIF) [file pone.0136290.s004.tif]

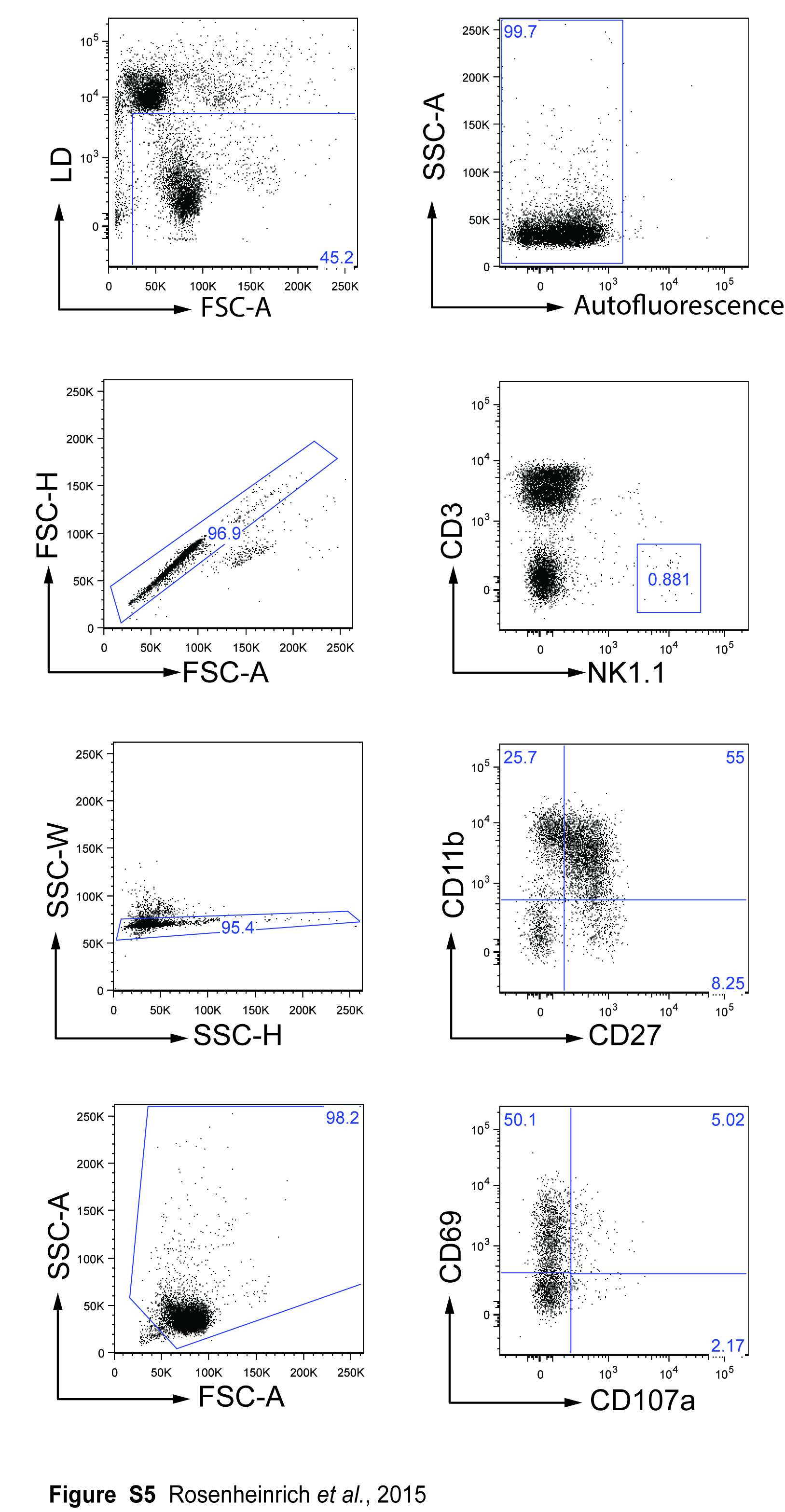

Supplement: S5 Fig — Three days post infection mLNs were isolated and stained with the following markers to differentiate NK cell subsets: Live/Dead (LD), CD3, NK1.1, CD11b, CD27, CD69, CD107a. After gating for living cells and doublet exclusion NK cells (CD3- NK1.1+) were further analyzed for their expression of CD11b and CD27. Subsets were defined a seither CD11b+/- and/or CD27+/-. CD11b+ CD27+ cells underwent additional analysis of their CD69 and CD107a expression. (TIF) [file pone.0136290.s005.tif]

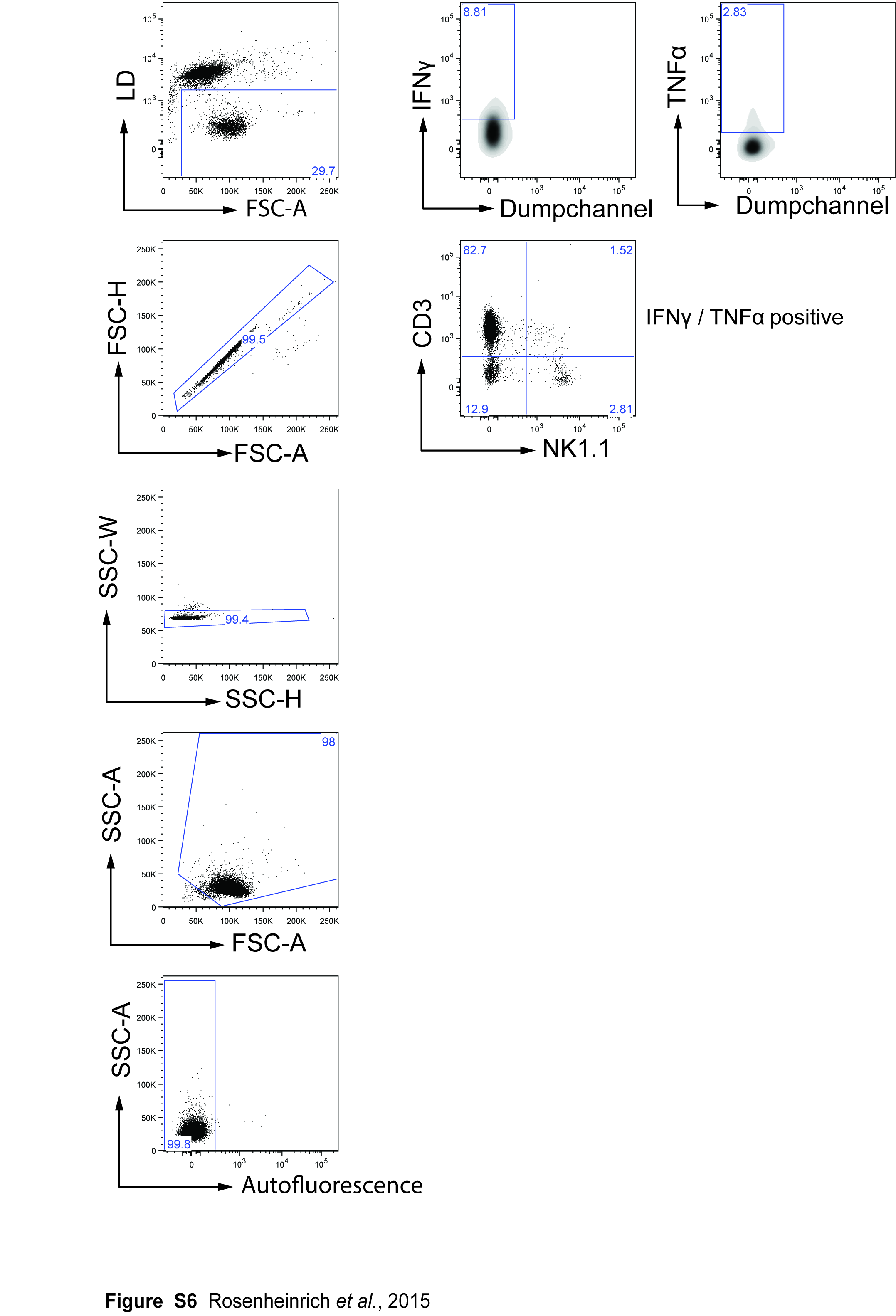

Supplement: S6 Fig — Three days post infection mLNs were isolated and stained with the following markers to differentiate cytokine producers: Live/Dead (LD), CD3, NK1.1, IFNγ and TNFα. Cells were first analyzed for their expression of the respective cytokine and afterwards the producing cells were associated with either CD3 for T-cells, NK1.1 for NK cells or expression of neither (of non T-cell, non NK cell origin)/ both (NKT cells). (TIF) [file pone.0136290.s006.tif]

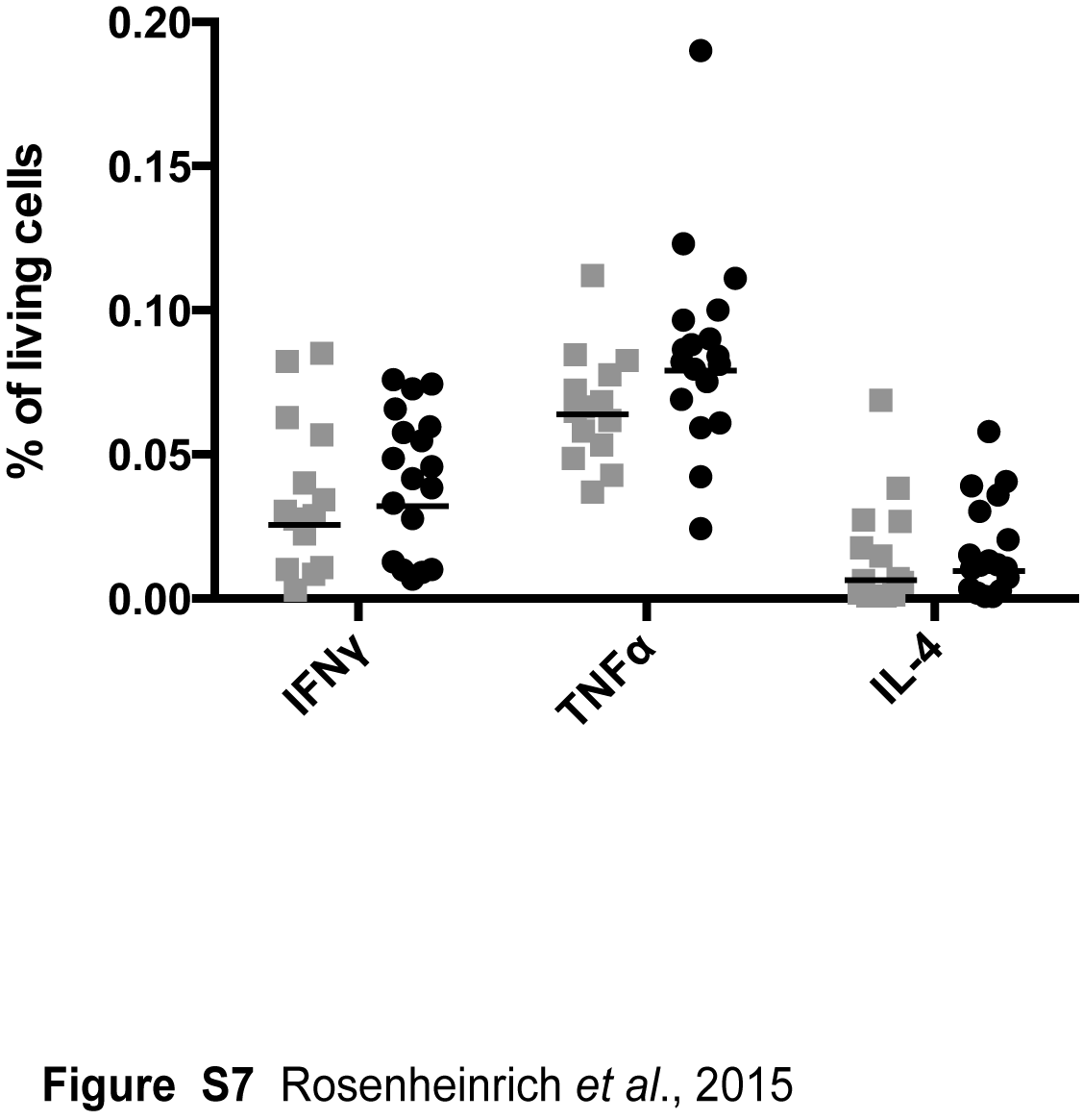

Supplement: S7 Fig — Three days post infection mLNs were isolated and stained with the following markers to differentiate cytokine producers: Live/Dead (LD), CD3, NK1.1, IFNγ, IL-4 and TNFα. Cells were analyzed for their expression of NKT surface markers (CD3+ NK1.1+). Subsequently, expression of the respective cytokines was investigated. (TIF) [file pone.0136290.s007.tif]
